# Combined Inhibition of Bcl2 and Bcr-Abl1 Exercises Anti-Leukemia Activity but Does Not Eradicate the Primitive Leukemic Cells

**DOI:** 10.3390/jcm10235606

**Published:** 2021-11-29

**Authors:** Michele Massimino, Paolo Vigneri, Stefania Stella, Elena Tirrò, Maria Stella Pennisi, Laura Nunziatina Parrinello, Calogero Vetro, Livia Manzella, Fabio Stagno, Francesco Di Raimondo

**Affiliations:** 1 Department of Clinical and Experimental Medicine, University of Catania, 95124 Catania, Italy; vigneripaolo@gmail.com (P.V.); stefania.stel@gmail.com (S.S.); ele_tir@yahoo.it (E.T.); perny76@gmail.com (M.S.P.); manzella@unict.it (L.M.); 2 Center of Experimental Oncology and Hematology, A.O.U. Policlinico “G. Rodolico-S. Marco”, 95123 Catania, Italy; 3 Division of Hematology, A.O.U. Policlinico “G. Rodolico-S. Marco”, 95123 Catania, Italy; lauraparrinello@tiscali.it (L.N.P.); gerovetro@gmail.com (C.V.); fsematol@tiscali.it (F.S.); diraimon@unict.it (F.D.R.)

**Keywords:** BCL2, BCR-ABL1, stem cell, CML, ALL, Venetoclax, Nilotinib, LTC-IC

## Abstract

Background: The management of Philadelphia Chromosome-positive (Ph+) hematological malignancies is strictly correlated to the use of BCR-ABL1 tyrosine kinase inhibitors (TKIs). However, these drugs do not induce leukemic stem cells death and their persistence may generate a disease relapse. Published reports indicated that Venetoclax, a selective BCL2 inhibitor, could be effective in Ph+ diseases, as BCL2 anti-apoptotic activity is modulated by BCR-ABL1 kinase. We, therefore, investigated if BCL2 inhibition, alone or combined with Nilotinib, a BCR-ABL1 inhibitor, affects the primitive and committed Ph+ cells survival. Methods: We used Ph+ cells isolated from leukemic patients at diagnosis. To estimate the therapeutic efficacy of BCL2 and BCR-ABL1 inhibition we employed long-term culture, proliferation and apoptosis assay. Immunoblot was used to evaluate the ability of treatment to interfere with the down-stream targets of BCR-ABL1. Results: Blocking BCL2, we observed reduced proliferation and clonogenic potential of CML CD34-positive cells and this cytotoxicity was improved by combination with BCR-ABL1 inhibitor. However, BCL2 inhibition, alone or in combination regiment with BCR-ABL1 inhibitor, did not reduce the self-renewal of primitive leukemic cells, while strongly induced cell death on primary Ph+ Acute Lymphoblastic Leukemia (ALL). Conclusion: Our results suggest that primitive CML leukemic cells are not dependent on BCL2 for their persistence and support that committed CML and Ph + ALL cells are dependent by BCL2 and BCR-ABL1 cooperation for their survival. The antileukemic activity of BCL2 and BCR-ABL1 dual targeting may be a useful therapeutic strategy for Ph+ ALL patients.

## 1. Introduction

The chromosome translocation (t9;22) forming the Philadelphia Chromosome (Ph+) generates the chimeric BCR-ABL1 oncogene, which triggers the Chronic Myeloid Leukemia (CML) and the Ph+ Acute Lymphoblastic Leukemia (Ph+ ALL). BCR-ABL1 oncogene encodes for an oncoprotein with constitutive tyrosine kinase activity causing stem cells transformation and leukemic clone expansion [1]. ABL-directed inhibitors, commonly named tyrosine kinase inhibitors (TKIs), represented by first-generation Imatinib (IM), second-Nilotinib, Dasatinib (DAS), Bosutinib (BOS) and third-generation Ponatinib (PON), are the first line treatment for CML and Ph+ B-ALL patients, inducing complete hematological, molecular and cytogenetic responses [2,3].

Although TKIs are effective in the eradication of committed Ph+ cells, they fail to induce leukemic stem cells eradication, hence, the persistence of this cell population may cause disease progression. Furthermore, Ph+ stem cells can accumulate several BCR-ABL1-dependent or -independent molecular alterations providing a reservoir generating a drug-resistance clone. In fact, about 50% of CML and most of Ph+ ALL patients show a transient benefit from these drugs, hence, curing Ph+ diseases with TKIs is rare and new therapeutic approaches are requested [4,5,6]. Drug-resistance clones have also been observed in acute myeloid leukemia and myelodysplastic syndromes, following anti-tumor therapies with hypomethylating agents [7].

The mitochondrial BCL2 family proteins consist of both anti-apoptotic and pro-apoptotic members. Dependent on their structure the BCL2 family is classified in three groups including (i) BCL2, BCL-xL and BCL-w exercising anti-apoptotic effect, (ii) Bax and Bak having a pro-apoptotic role and (iii) Bik and Bid which display pro-apoptotic functions as well. Hence, BCL2 family proteins play a critical role in the survival of leukemic cells [8,9]. Different authors report that BCL2 protein is consistently expressed in normal stem cells and that these high levels are maintained after BCR-ABL1-mediated transformation [10,11]. Published data indicate that BCR-ABL1 exercises its pro-survival activity by increasing BCL2 family proteins expression [12,13]. On the basis of these observations, the BCL2 inhibition alone or combined with a BCR-ABL1 inhibitor might represent a useful strategy to eradicate the leukemic cells.

A selective BCL2 inhibitor, ABT-199 known as Venetoclax, is demonstrated to have a strong antileukemic effect against several hematological malignances such as chronic lymphocytic leukemia, acute myeloid leukemia and in-vitro and in-vivo CML models where this cytotoxicity improves the BCR-ABL1 inhibitors efficacy [14,15,16,17,18,19]. To support these results, two clinical studies are ongoing (NCT02689440, NCT02115295) in order to evaluate the efficacy of Venetoclax in combination with other drugs in CML patients in chronic or blast phase.

We investigated the effect of BCL2 inhibition by Venetoclax alone or combined with Nilotinib [19] in cells derived from chronic phase CML (CP-CML) and Ph+ ALL patients expressing p210 and p190 BCR-ABL1 isoforms, respectively. Using long-term culture-initiating cells (LTC-IC), cell death analysis, clonogenic and proliferation assays we determined the role of BCL2 in the BCR-ABL1-mediated survival activity. Furthermore, we analyzed the effect of this regimen on BCR-ABL1 down-stream targets STAT5 and CRKL proteins [3].

We demonstrate that BCL2 inhibition, alone or combined with Nilotinib have been effective on CML CD34-positive progenitors and Ph+ B-ALL cells but failed to interfere with self-renewal properties of CML primitive cells.

## 2. Materials and Methods

### 2.1. Patients

Chronic Phase CML patients (p210 CP-CML, *n* = 4) and Ph+ B-ALL patients (p190 B-ALL, *n* = 3) were diagnosed by real-time PCR as previously reported [20]. For B-ALL patients’ peripheral neoplastic cells expressing > 80% CD19-positive were eligible for this study. All patients were followed in the Division of Hematology of the A.O.U. Policlinico–G. Rodolico-S. Marco and signed an informed consent releasing anonymously their samples for research purposes in accordance with the Declaration of Helsinki.

### 2.2. Isolation and Culture of Ph+ Primary Cells

CD34-positive cells were immunomagnetically separated from aspirated bone marrow of CML patients at diagnosis as previously published [21] and grown in presence of low cytokines concentration (FLT3 ligand 5 ng/mL, stem cell factor 5 ng /mL, IL-3 and IL-6 1 ng/mL, all from Stem Cell technologies) to avoid impairing BCR-ABL1-dependent proliferation. To isolate Ph+ B-ALL cells we used peripheral blood of patient at diagnosis showing 80% of lymphoblastic cells obtained by gradient separation using Ficoll Paque Premium, cultivated in RPMI supplemented with 10% of non-inactivated fetal bovine serum (FBS) (EuroClone), 2 mM of glutamine, 100 µg/mL and 50 µg/mL of streptomycin (all from Sigma-Aldrich). Nilotinib was provided by Novartis while Venetoclax was purchased from Santa Cruz.

### 2.3. Drug Treatment

Cells were exposed to Nilotinib and Venetoclax, alone or in combination, at concentration of 2 µM [22] and 400 nM [19], respectively. For all experiments, cells were exposed to drug treatment for 24 h.

### 2.4. Trypan Blue Exclusion Assay

Cells were counted by mixing 10 µL of each cell suspension with 10 µL of 0.4% of trypan blue solution and their number determined in a hemocytometer.

### 2.5. Long Term Culture-Initiating Cells (LTC-ICs) Assays

LTC-IC frequency was calculated by Limiting Dilution Analysis (LDA). As feeder layers in 96-well plates, 1.5 × 10^4^ M2-10B4 mouse fibroblast (Stem Cell Technologies, Vancouver CAN) were established and blocked for 24 h with 2 µg/mL Mytomicin C (Sigma Aldrich, St Louis USA) [23]. Bone morrow CML CD34-positive progenitors were pretreated or not for 24 h with Nilotinib, Venetoclax or their combination. At this time were co-cultivated on feeder cells using 40 replicates of two-fold cell dilutions (from 50 to 400 initial test cells) in long-term culture medium (MyeloCult H5100 from StemCell Technologyes) for 5 weeks with weekly half-medium changes. After 5 weeks, cells were overlaid with methylcellulose (Methocult H4435, StemCell Technologies) supplemented with conditioned medium derived from 5637 cells [24]. Colonies were counted under the microscope after two additional weeks. LTC-IC frequency was calculated using the L-Calc software (StemCell Technologies) [25,26,27]. Number of LTC-IC was obtained by ratio between the number of initial cells tested used in bulk analysis and the LTC-IC frequency value calculated by LDA analysis [25,28].

LTC-IC-derived CFUs were measured performing LTC-IC assays bulk analysis. A total of 5 × 10^4^ CD34-positive initial tested cells were cultivated on 445 × 10^3^ M2-10B4 feeder cells, treated as described above, in a 35 mm dish. After 5 weeks, adherent and non-adherent cells were collected and 5 × 10^4^ hematopoietic cells were resuspended in methylcellulose (H4435) as previously described [25,26,27,29,30]. LTC-IC-derived CFUs were expressed as the number of clonogenic progenitors obtained after 15 days of methylcellulose culture (number of colonies multiplied by the total number of hematopoietic cells counted after 5 weeks of culture) divided by the number of initial test cells seeded on fibroblasts. LTC-IC division rate was obtained from ratio between the LTC-IC-derived CFUs and the number of LTC-IC [25].

### 2.6. Colony Forming Unit (CFUs) and Secondary Re-Plating (CFUs-r) Assays

For colony forming units (CFUs) and secondary re-plating assays (CFUs-r), 500 CML bone marrow CD34-postive cells, untreated or exposed to Nilotinib, Venetoclax or in combination regiment for 24 h, were seeded in methylcellulose medium (Methocult H4435, StemCell Technologies). Total myeloid colonies were counted after 15 days of culture for CFUs. For secondary re-plating the total CFUs-r were counted and collected after 10 days. The methylcellulose was dissolved in 2 mL of Iscove’s D-MEM supplemented with 2% FBS. Cells were then centrifuged at 1200 RPM for 10 min at room temperature, and 1 × 10^4^ cells re-implanted (secondary re-plating) in methocult as above. The colonies were counted after 15 days [31,32]. For all colonies count an optical microscope (Olympus IX71) was used.

### 2.7. Western Blotting, Immunoblotting and Densitometric Analysis

In total, 3 × 10^5^/mL CD34-positive and 1 × 10^6^/mL B-ALL cells were left untreated or exposed to Nilotinib, Venetoclax or their combination for 24 h. Cells were then lysed in Laemmli buffer [62.5 mM Tris-HCl (pH 6.8), 2% *w*/*v* SDS, 10% glycerol, 50 mM dithiothreitol (DTT), 0.01% *w*/*v* bromophenol blue], sonicated, denaturated and each protein lysate was separated by SDS-PAGE. Proteins were transferred on nitrocellulose membranes which were hybridized using the following antibodies: anti-phospho-CRKL (Tyr207) (clone 3181), anti-CRKL (clone 32H4), anti-STAT5 (clone 94205) and anti-pSTAT5 (Y694) (clone 9351) (all from Cell Signaling); anti-Actin from Sigma-Aldrich. After incubation with primary antibodies, appropriate horseradish peroxidase conjugated secondary antibodies (Amersham Biosciences) were added and proteins were then detected using the enhanced chemiluminescence (ECL) reagent Star (Euroclone) or WesternSure PREMIUM (Li-cor). Chemiluminescent images were digitally captured on the c-Digit blot scanner and a densitometric analysis was performed using the Image J software. For each protein, relative densitometric units were obtained normalizing for actin. The final relative densitometric units were obtained by calculating the ratio between phosphorylated versus total protein fractions.

### 2.8. Cell Death Assay

A total of 3 × 10^5^/mL CD34-positive and 1 × 10^6^/mL Ph+ B-ALL cells were exposed or no to Nilotinib, Venetoclax or in combination regiment for 24 h. At this time cells were stained by Annexin V/7-AAD (Beckman Coulter, Brea, CA, USA) and analyzed by cytofluorimetric analysis employing Cytomics FC500.

### 2.9. Statistical Analysis

Statistical significance was calculated using the Prism Software version 8.0 applying analysis of variance (ANOVA) plus Bonferroni’s posttests.

## 3. Results

### 3.1. BCL2 and BCR-ABL1 Kinase Dual Inhibition Exercises Anti-Clonogenic and Anti-Proliferation Activity on Committed CP-CML Progenitors

**Figure 1:**
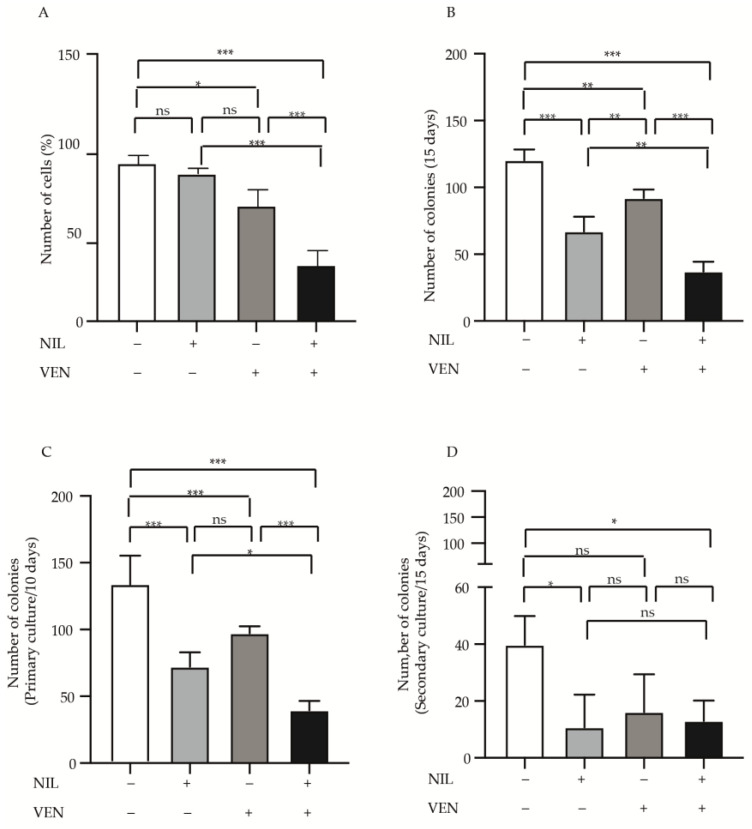
BCR-ABL1 kinase inhibition improves the anti-proliferative and anti-clonogenic activity of Venetoclax in committed CML CD34-positive cells. (**A**) Bone marrow CD34-positve cells derived from CML patients (*n* = 4) at diagnosis, were exposed for 24 h to the indicated drugs (Venetoclax 400 nM, Nilotinib 2 μΜ). Histograms report the viability of cells obtained setting arbitrary at 100% the number of cells implanted at the start of the experiment. (**B**) The same CD34-positive progenitors, treated as reported in (**A**), were implanted in methylcellulose in the presence of the same drugs regimen. Histograms report the number of colonies obtained after 15 days of culture. (**C**,**D**) CD34-positive progenitors treated as reported in (**B**) was used for primary (**C**) and secondary (**D**) plating performed as indicated in method. Histo-grams show the number of colonies counted after 10 (**C**) and 15 (**D**) days of culture. For all experiments, bars indicate the standard deviation derived from two experiments performed in duplicate. Statistical significance was calculated by analysis of variance (ANOVA) plus Bonferroni’s posttests (*p* < 0.05 *, *p* < 0.01 **, *p* < 0.001 ***). UT = Untreated, VEN = Venetoclax, NIL = Nilotinib.

**Figure 2:**
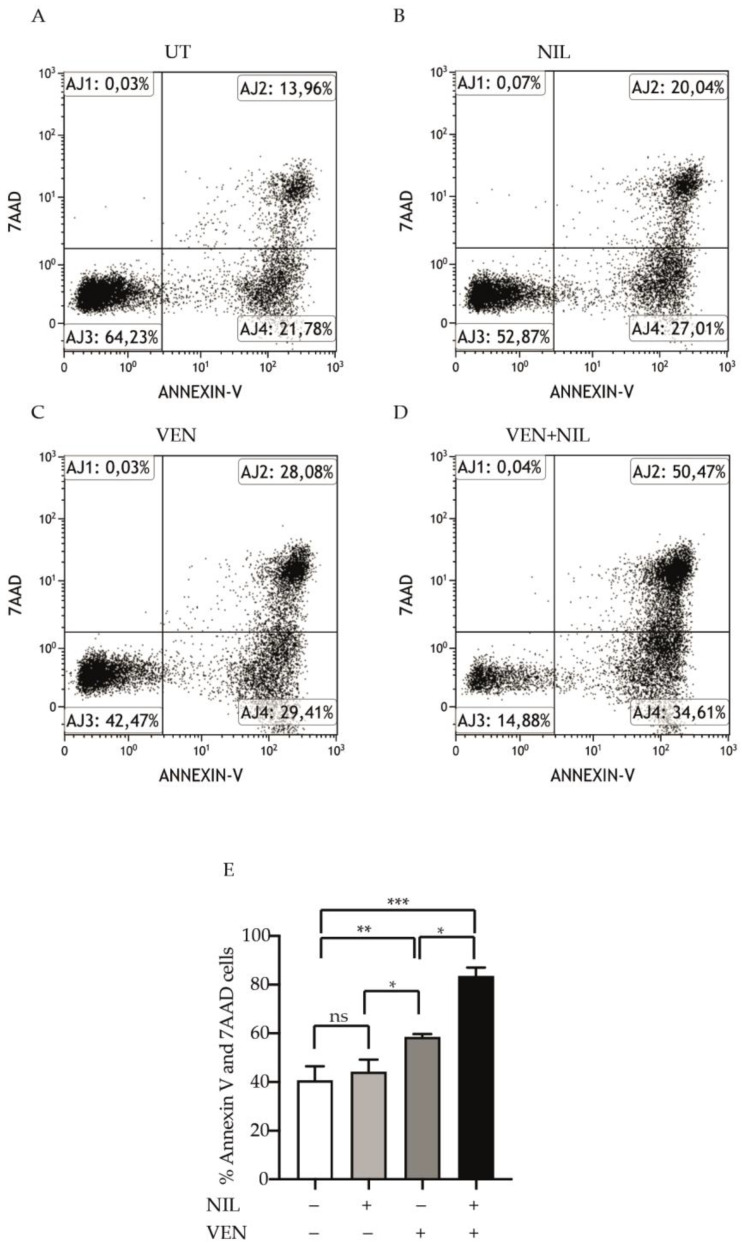
Pro-apoptotic effect of BCL2 and BCR-ABL1 dual targeting in committed CML CD34-positve cells. (**A**–**D**) Representative experiment of bone marrow CD34-progenitors untreated (**A**) or treated with Nilotinib (2 μM) (B), Venetoclax (400 nM) (**C**) or their combination (**D**). After 24 h cells were collected and stained with Annexin V/7-AAD. Scatter plots indicate the unstained (AJ3) and Annexin V (AJ4), Annexin V/7-AAD (AJ2) or 7-AAD (AJ1) stained cells. (**E**) Histograms report the average percentage of Annexin and 7-AAD cells obtained from CML patients (*n* = 3). Bars indicate the standard deviation resulting from average of three patients. Statistical significance was calculated by analysis of variance (ANOVA) plus Bonferroni’s posttests (*p* < 0.05 *, *p* < 0.01 **, *p* < 0.001 ***). UT: Untreated, NIL: Nilotinib, Ven: Venetoclax.

To investigate the cytotoxic activity of BCL2 and BCR-ABL1 kinase inhibition, we exposed CP-CML CD34-positive progenitors to Venetoclax and Nilotinib or their combination for 24 h (Figure 1). The treatment with Nilotinib did not reduce cell number (fold reduction 1.07), while Venetoclax showed a significant but modest effect (fold reduction 1.35, *p* < 0.05). Interestingly, we observed that Venetoclax increased the Nilotinib cytotoxicity, strongly reducing the number of committed CP-CML cells (fold reduction UT, Nilotinib and Venetoclax vs. Nilotinib + Venetoclax 2.7, 2.6 and 2.2, respectively, *p* < 0.001) (Figure 1A). Different results were observed when we performed the colony-forming unit assay. Compared to the untreated condition, although with less efficacy than Nilotinib, Venetoclax reduced the clonogenic potential of committed CP-CML cell (fold reduction Nilotinib 1.8 *p* < 0.001, Venetoclax 1.3 *p* < 0.01). In turn, Nilotinib + Venetoclax regimen was more potent than two drugs alone, inhibiting the colonies formation of 3.2 (*p* < 0.001), 1.8 (*p* < 0.01) and 3 (*p* < 0.001) folds when compared to untreated, Nilotinib or Venetoclax alone, respectively (Figure 1B). Next, we wanted to evaluate if Venetoclax alone or combined with Nilotinib interferes with the ability of CP-CML progenitors to perpetuate the colonies formation. To this end, we performed a primary and secondary re-plating in methylcellulose. For primary plating the colonies were counted after 10 days (Figure 1C), observing comparable data reported in Figure 2B. When we counted the colonies obtained after secondary re-plating, we detected a significant cytotoxicity by Nilotinib (fold 3.7, *p* < 0.05) and Nilotinib + Venetoclax (fold 3, *p* < 0.05) but not by Venetoclax (fold 2.4) (Figure 1D). Overall, Venetoclax + Nilotinib combination was significantly more potent than the drugs alone, with the exception of secondary replating experiment. All together these findings support that Venetoclax + Nilotinib combined treatment eliminates the committed CML cells, while the more primitive cells were less sensitive to BCL2 and BCR-ABL1 kinase inhibition.

### 3.2. BCL2 and BCR-ABL1 Kinase Co-Targeting Kills Committed CP-CML Progenitors

In order to investigate if Venetoclax- or Venetoclax + Nilotinib-mediated cytotoxicity was dependent on apoptosis induction, we exposed CP-CML CD34-positive progenitors to Nilotinib, Venetoclax or their combination for 24 h and then we stained them by Annexin V and 7AAD (Figure 2A–D). Compared to untreated cells, Venetoclax was significantly more potent than Nilotinib to kill leukemic cells (Venetoclax: 58.5%, *p* < 0.01, Nilotinib: 44.3%) and Nilotinib + Venetoclax combination was strongly cytotoxic (Nilotinib + Venetoclax:83.4%, *p* < 0.001) (Figure 2E) showing a cooperative effect. Hence, these results indicate that the BCL2 and BCR-ABL1 kinase co-inhibition exercises a potent pro-apoptotic effect on committed CP-CML cells.

### 3.3. BCL2 Inhibition Does Not Affect the Activity of BCR-ABL1-Dependent Pro-Survival Mediator STAT5

**Figure 3:**
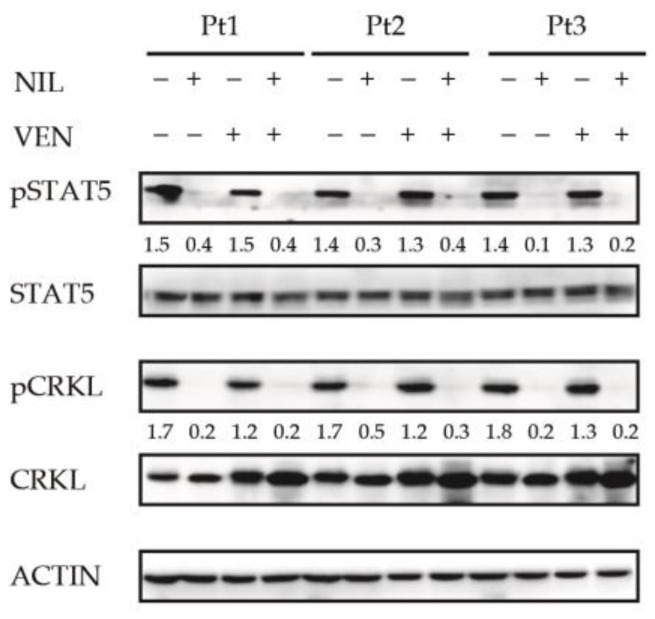
BCL2 and BCR-ABL1 co-targeting reduces the STAT5 and CRKL phosphorylation levels in committed CML CD34-positive cells. A Bone marrow CD34-positve progenitors (CML patients *n* = 3) were exposed to the indicated drugs (Venetoclax 400 nM, Nilotinib 2 μM) and derived cell lysates separated by SDS-PAGE. Nitrocellulose membrane was hybridized with specified antibodies. Actin was used as loading control. The numbers indicate the densitometric analysis obtained using Image J software. VEN = Venetoclax, NIL = Nilotinib.

To investigate if the antileukemic activity of the BCL2 inhibition was dependent on its ability to interfere with BCR-ABL1-dependent pro-survival mediator STAT5, we analyzed its phosphorylation level using CRKL as control of the BCR-ABL1 suppression activity [24]. Relative densitometric units revealed STAT5 and CRKL phosphorylation reduction induced only by Nilotinib (fold 37.4) or its combination with Venetoclax (fold 37.5) (Figure 3) suggesting that this event was dependent on Nilotinib-mediated BCR-ABL1 kinase inhibition.

These results suggest that antileukemic activity mediated by Venetoclax is not dependent on BCR-ABL1 signaling alteration in CML progenitors.

### 3.4. Primitive CP-CML Cells Are Not Dependent on BCL2 Antiapoptotic Activity for Their Survival

**Figure 4:**
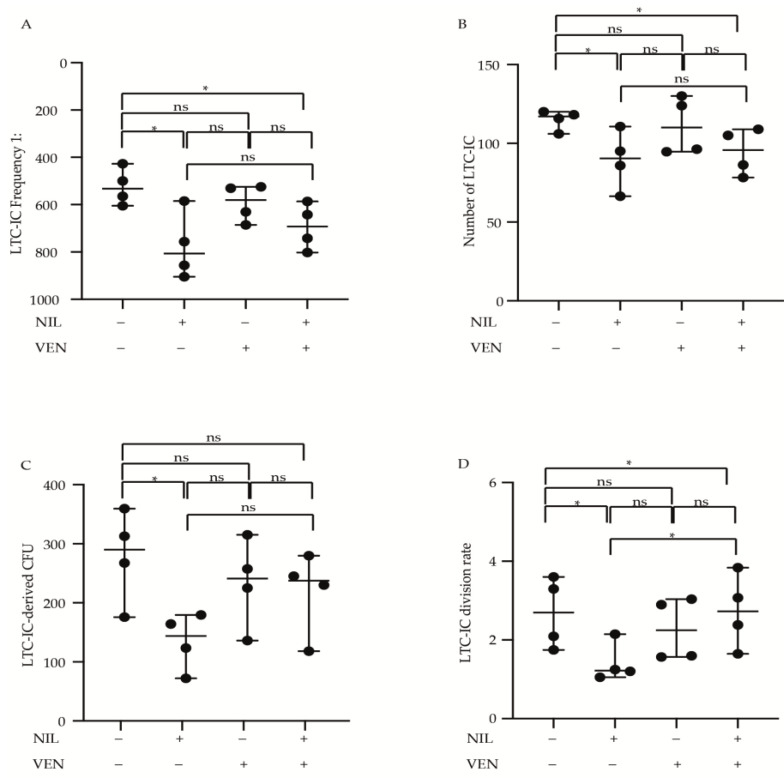
BCL2 and BCR-ABL1 kinase inhibition does not eliminate the primitive CML population. The scatter plot shows the LTC-IC frequency (**A**), number of LTC-IC (**B**), LTC-IC-derived CFU (**C**) and LTC-IC division rate (**D**) of the bone marrow primitive CD34-positive cells derived from CML patients (*n* = 4) at diagnosis (Venetoclax 400 nM and Nilotinib 2 μΜ). Data are reported as median with range obtained from each experiment performed in duplicate. Statistical significance was calculated by unpaired one-tailed t-tests with 95% confidence intervals (*p* < 0.05 *). UT = Untreated, VEN = Venetoclax, NIL = Nilotinib.

Previously, data demonstrated that BCL2 and BCR-ABL1 kinase dual inhibition eradicates CML stem cells derived from patients in blast crisis [19]. We investigated the cytotoxic effects of BCL2 and BCR-ABL1 kinase inhibition by Venetoclax and Nilotinib, alone or in combination, on self-renewal properties of primitive CP-CML cells. Using LDA assay we observed that Venetoclax was not able to reduce the LTC-IC frequency, (untreated = 1:532, Venetoclax = 1:580), and although its combination with Nilotinib reduced the frequency of leukemic cells significantly, (Venetoclax + Nilotinib = 1:692, *p* < 0.05) this effect was less potent then Nilotinib alone (1:806, *p* < 0.05) (Figure 4A). Moreover, comparing to untreated condition, while Nilotinib + Venetoclax combination reduced equaling the LTC-IC absolute number than Nilotinib alone (LTC-IC number untreated = 117, Venetoclax + Nilotinib = 96, *p* < 0.05, Nilotinib = 91, *p* < 0.05), Venetoclax failed in this effect (LTC-IC number Venetoclax = 110) (Figure 4B). Subsequently, we measured the clonogenic and division rate of CP-CML primitive leukemic cells by bulk analysis (Figure 4C, D). We detected a significant reduction in both LTC-IC-derived CFUs and division rate after Nilotinib treatment (*p* < 0.05), while no significant cytotoxicity was observed after Venetoclax alone or combined with Nilotinib (LTC-IC-derived CFUs untreated = 290, Nilotinib = 143, Venetoclax = 241, Venetoclax + Nilotinib = 237) (Figure 4C) (Division rate untreated = 3.4, Nilotinib = 1.5, *p* < 0.05, Venetoclax = 2.8, Venetoclax + Nilotinib = 3.4, *p* < 0.05) (Figure 4D).

All together these data support the hypothesis that primitive CP-CML cells are not dependent on BCL2 protein for their survival as the self-renewal reduction observed after BCL2 and BCR-ABL1 kinase inhibition was dependent only by Nilotinib exposure.

### 3.5. BCR-ABL1-Positive B-ALL Cells Are Sensitivity to BCL2 Inhibition

**Figure 5:**
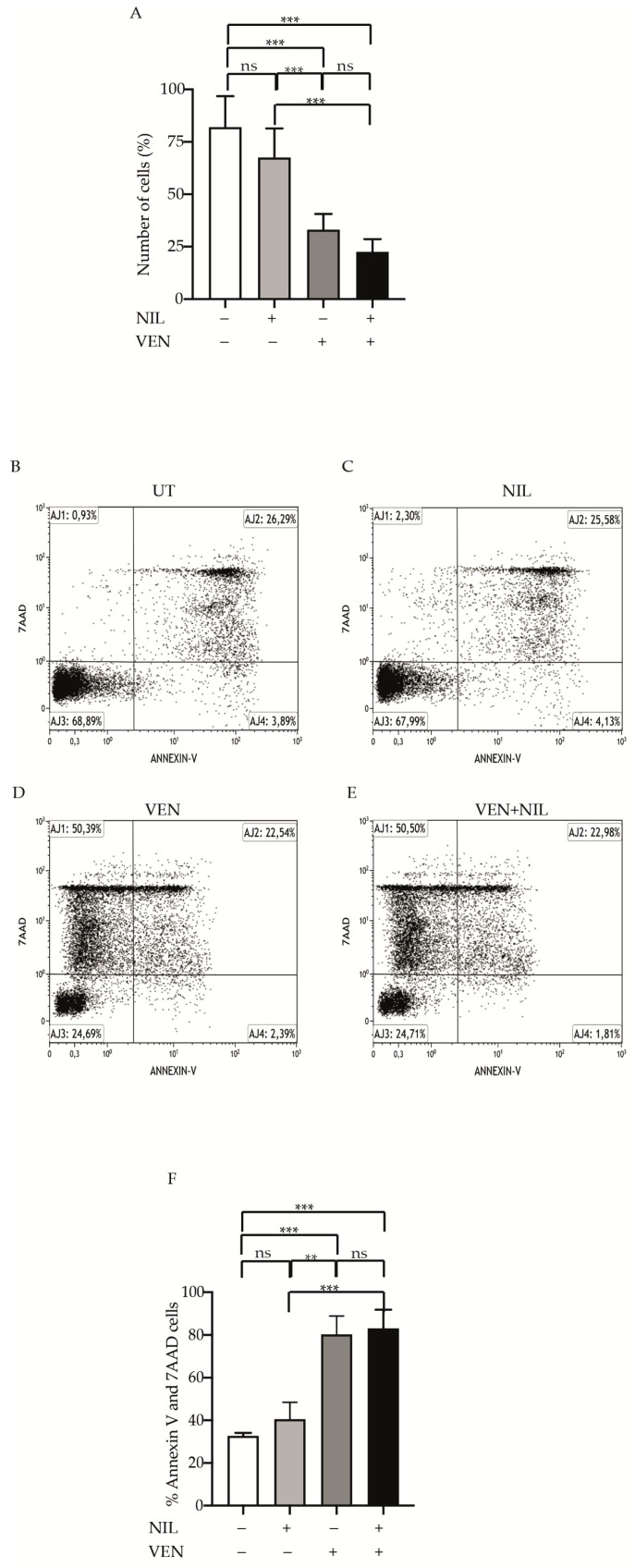
Anti-proliferative and pro-apoptotic activity of BCL2 and BCR-ABL1 co-inhibition in Ph+ lymphoblastic B-ALL cells. (**A**). Peripheral B-ALL cells derived from B-ALL patients (*n* = 3) at diagnosis were exposed for 24 h to the indicated drugs (Venetoclax 400 nM, Nilotinib 2 μM). Histograms report the viability cells obtained setting arbitrary at 100% the number of cells implanted at the start of the experiment. (**B**–**E**) Representative experiment of peripheral B-ALL cells untreated (**B**) or treated with Nilotinib (**C**), Venetoclax (**D**) or their combination (**F**). After 24 h cells were collected and stained with Annexin V/7-AAD. Scatter plots indicate the unstained (AJ3), Annexin V (AJ4), Annexin V/7-AAD (AJ2) or 7-AAD (AJ1) stained cells (*n* = 2). F Histograms report the average percentage of Annexin and 7-AAD cells obtained from B-ALL patients. Bars indicate the standard deviation resulting from the average of two patients. Statistical significance was calculated by analysis of variance (ANOVA) plus Bonferroni’s posttests (*p* < 0.01 **, *p* < 0.001 ***). UT:Untreated, NIL:Nilotinib, Ven: Venetoclax.

To evaluate if BCL2 and BCR-ABL1 inhibition was effective against Ph+ B-ALL cells, we exposed them to Nilotinib, Venetoclax or their combination, measuring both proliferation and cell death (Figure 5). Although statistically not significant, compared to untreated cells, Nilotinib reduced the number of cells after 24 h (fold 1.5) while Venetoclax and Venetoclax + Nilotinib were more potent in this effect, showing a statistically significant reduction of 3 (*p* < 0.001) and 4.5 (*p* < 0.001) fold, respectively (Figure 5A). Staining the cells by Annexin V and 7AAD (Figure 5B–E), we observed a not statistically significant cell death after Nilotinib exposure (staining 40.15%) comparable to untreated condition (Figure 5F). Interestingly, when we exposed the cells to Venetoclax we found a statistically significant increase in stained cells (staining 77.7%, *p* < 0.001), weakly improved by Nilotinib combination (staining 83.15%, *p* < 0.001). Hence, these finding support an important implication suggesting that BCL2 protein plays a critical role in Ph+ B-ALL cells survival.

### 3.6. BCL2 Targeting Exercises Antileukemic Activity by Phosphorylation Reduction in Pro-Survival Factor STAT5 in Ph+ Positive B-ALL Cells

**Figure 6:**
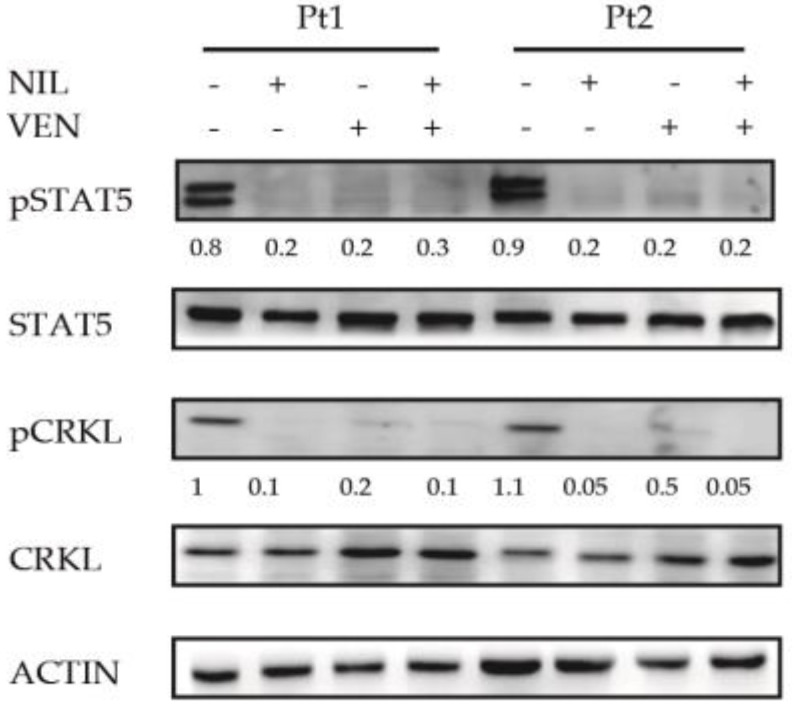
Single or combined BCL2 and BCR-ABL1 inhibition represses the STAT5 and CRKL phosphorylation in Ph+ B-ALL cells. Peripheral blood lymphoblastic cells derived from Ph+ B-ALL patients (*n* = 2) were exposed to the indicated drugs (Venetoclax 400 nM, Nilotinib 2 μΜ). Cell lysates were separated by SDS-PAGE and nitrocellulose membrane hybridized by specified antibodies. Actin was used as loading control. The numbers indicate the densitometric analysis obtained using Image J software. VEN = Venetoclax, NIL = Nilotinib.

Subsequently, we analyzed the impact of the BCL2 inhibition on BCR-ABL1 downstream targets STAT5 in Ph+ B-ALL cells using CRKL as control for BCR-ABL1 kinase suppression [24] (Figure 6). We used densitometric analysis to detect the differences in the phosphorylation levels of STAT5 and CRKL. As expected, Nilotinib reduced the phosphorylation levels of STAT5 (4.3- and 4.2- fold in pt1 and pt2) and CRKL (fold reduction of 10 and 22 for pt1 and pt2) compared to untreated cells. Unexpectedly, we observed that BCL2 inhibition alone reduced the phosphorylation levels of both STAT5 (4-fold in pt1 and 4.5-fold in pt2) and CRKL (5- and 2.5-fold in pt1 and pt2). Furthermore, this effect was maintained after its combination with Nilotinib for both STAT5 (fold reduction of 2.6 and 4.2 for pt1 and pt2) and CRKL (fold reduction of 10 and 25 for pt1 and pt2) proteins. These results highlight the relationship between BCR-ABL1 downstream targets and BCL2 activity in Ph+ B-ALL cells.

## 4. Discussion

Although the introduction of ABL-directed inhibitors, commonly named TKIs, induced long-term survival of CML patients, about 50% of them and patients affected by Ph+ B-ALL do not completely benefit from these drugs, often requiring an additional therapeutic approach [4]. The reasons of this failing are related to the development of BCR-ABL1-dependent [33,34,35,36] or -independent [37,38,39,40] resistance mechanisms as well as the “non-oncogene addicted” property shown by leukemic stem cells [41,42]. Hence, on the basis of the concept of precision medicine, which increased the survival of patients affected by different tumor types [43,44,45,46,47,48,49] the identification of new therapeutic targets, able to overcome the TKIs inefficacy, may result in useful strategies for patients failing the conventional therapy.

Different authors demonstrated the cytotoxic effect of BCL2 inhibition in Ph- [10,50,51,52] and Ph+ [19,53,54,55] leukemia cells. Furthermore, we have previously reported that Venetoclax alone or combined with Nilotinib was strongly cytotoxic in primary Ph+ ^p210^B-ALL cells [56]. Hence, all together these data demonstrate a direct involvement of BCL2 protein in leukemogenesis, also confirming published data reporting that BCL2-mediated antiapoptotic effect is regulated by BCR-ABL1 kinase [12,57]. Despite these results, other authors demonstrated that BCR-ABL1-dependent or -independent resistance mechanisms might not be driven by BCL2-mediated antiapoptotic effect [58] and the role of BCL2 protein in the leukemic transformation and drug resistance in Ph+ cells is not clear.

In this work we analyzed the cytotoxic effect of BCL2 inhibition by Venetoclax alone or in combination regimen with Nilotinib in primitive and committed CP-CML as well as in Ph+ B-ALL cells. We observed that Venetoclax was able to kill committed CML precursors, but not to interfere with the self-renewal properties of more primitive CP-CML cells, an effect that was observed after Nilotinib exposure [24] and, though less marked, after Venetoclax + Nilotinib combination. Although these data are in contrast with those reported by Carter et al. [19], it is possible that this discordance is dependent on different used models. In fact, Carter used a mouse model to mimic human CP-CML and the primitive leukemic cells were derived from CML patients in blast crisis, while our experiments were conducted on cells collected from patients in chronic phase. Furthermore, the reduced efficacy of BCL2 inhibition on more primitive leukemic cells, was confirmed when we performed a secondary replating experiment, establishing thus that this therapeutic approach does not alter the BCR-ABL1-mediated survival in these cells. Hence, CML disease status may reflect a diverse intracellular network responsible for different responses to BCL2 inhibition alone or combined with BCR-ABL1 kinase inhibitor. Furthermore, we detected that Nilotinib and Venetoclax combination did not reduce both number of LTC-IC CFU and division rate. We hypothesize that this phenomenon could be dependent on the primitive state of leukemic progenitors derived from long term culture.

The observation that, in committed CML cells, BCL2 inhibition induced apoptosis, also increasing the BCR-ABL1 kinase inhibitor efficacy, implicates the role of BCL2 in the BCR-ABL1-mediated pro-survival activity. However, this cytotoxicity was not dependent on modification of BCR-ABL1 downstream targets by BCL2 inhibition, suggesting that other pathways are involved in the anti-leukemic effect of Venetoclax.

Ph+ B-ALL cells are sensitive to different cytotoxic agents, but complete remissions are not durable, and up to 75% of patients show disease relapse. ABL-directed inhibitors, such as imatinib (IM), were proposed in combination therapy with different cytotoxic drugs to improve their effects [59]. Here, we report that the effect of Venetoclax might be different on Ph+ ALL cells compared to CML cells. We have confirmed the published data regarding high sensitivity of these cells to Venetoclax. BCL2 inhibition by Venetoclax, alone, is sufficient to induce cell death in Ph+ B-ALL cells and this effect was maintained and, also, weakly improved by Nilotinib combination. However, we observed a phosphorylation reduction in STAT5, indicating that, in contrast to CML, the antileukemic effect of Venetoclax, could be dependent on its ability to interfere with pro-survival intracellular signaling mediated by BCR-ABL1 and STAT5 in Ph+ B-ALL cells. Different authors reported direct and indirect correlation between BCL2 protein and STATs or CRKL in different tumor models [60,61,62,63,64,65,66,67], supporting, thus, our data.

Collectively, our preclinical results demonstrate that, although the committed CP-CML progenitors have been strongly sensitive to Venetoclax, CP-CML primitive leukemic cells do not dependent on BCL2 protein for their persistence. In committed CP-CML cells, the BCL2 and BCR-ABL1 kinase dual targeting demonstrated superior antileukemic activity, compared to either inhibition alone, suggesting that more differentiated leukemic cells are dependent on BCL2 and BCR-ABL1 kinase cooperation for their survival. On the contrary, in Ph+ B-ALL cells, BCL2 and BCR-ABL1 kinase inhibition showed strong antileukemic activity and this cytotoxic effect is mediated by the BCL2 inhibitor, Venetoclax. In conclusion, these results support that the antileukemic activity of BCL2 and BCR-ABL1 dual targeting does not eradicate the primitive leukemic cells in CP-CML patients but may be a useful therapeutic strategy for Ph+ ALL patients.
